# Intra-Operative Cochlear Nerve Function Monitoring in Hearing Preservation Surgery: A Systematic Review of the Literature

**DOI:** 10.3390/audiolres12060066

**Published:** 2022-12-15

**Authors:** Marzia Ariano, Sebastiano Franchella, Giulia Tealdo, Elisabetta Zanoletti

**Affiliations:** Section of Otorhinolaryngology-Head and Neck Surgery, Department of Neurosciences, University of Padova, 35128 Padova, Italy

**Keywords:** hearing-sparing surgery, cochlear nerve, monitoring

## Abstract

With the recent scientific and technical developments, hearing preservation surgery is becoming a growing objective in inner ear pathologies, especially for vestibular schwannomas. In this review, we aim to describe the pros and cons of the following cochlear nerve monitoring techniques: ABRs (auditory brainstem responses), DENM (direct eighth cranial nerve monitoring), EcochG (electrocochleography), CNAP (cochlear compound nerve action potentials), DPOAE (distortion product otoacoustic emissions), PAMRs (postauricular muscle responses). The Cochrane library, Scopus, DynaMed, and PubMed databases were screened to obtain any relevant papers from October 2009 to the present day. Due to the heterogeneity of the existing studies in the literature, there is no way to tell whether a technique is better than another. All authors reported satisfactory outcomes with the cochlear nerve monitoring techniques tested, either alone or in combination.

## 1. Introduction

With recent scientific and technical developments, hearing preservation surgery is becoming a growing objective in inner ear pathologies. In particular, more and more vestibular schwannomas (VSs) are being approached using the retrosigmoid or middle fossa approach, thus permitting, in specific situations, the preservation of hearing function.

The VS is a benign tumour originating from the Schwan’s sheath, with a slow pattern of growth [1]. It is a clinical concern because of its mass effect on the surrounding delicate anatomical structures, sometimes resulting in initial clinical presentations of tinnitus and hearing loss, or even deafness [2].

Recent technological developments, and in particular the increasing use and accuracy of magnetic resonance imaging (MRI), have increased the rate of detection of small acoustic neuromas, which are often asymptomatic. Currently, there are several management options for small acoustic neuromas. The wait-and-scan approach consists of observation with repeated radiological follow-up when the acoustic neuroma shows a slow/null pattern of growth [3]; nonetheless, in these cases, during observation time, irreversible hearing loss could still occur [4]. For small acoustic neuromas radiation therapy with Gamma Knife could also be considered, which is a less invasive option than surgical tumour removal. If this option is chosen, even if some authors report good functional outcomes with satisfactory rates of facial nerve function preservation in up to 95–100% of cases, the tumour is not eradicated, and control of disease needs to be assessed over the long term. Moreover, even if short/midterm hearing preservation associated with radiation therapy, in some papers, is reported to be up to 95%, there is still insufficient data in the literature to assess whether this outcome effectively lasts after a long follow up period [3,4,5]. Carlson et al., reported a 10-year rate of hearing preservation after radiotherapy of 23% [6].

The surgical approach in acoustic neuroma has gained increasing consensus since 1991, the year of the recommendations of the National Institutes of Health Consensus Development Conference on Acoustic Neuroma. Since then, microsurgery has been considered the best modality to treat this condition: the goals of this surgery have become progressively more advanced, with the aim to obtain both complete tumour removal and preserve neurological function of adjacent structures, including both facial and cochlear nerve function [1]. It is not surprising, then, that in experienced centres, more and more authors have chosen this therapeutic option. According to them, in fact, active treatment, compared to observation alone, offers a better chance of hearing preservation in the long term, enabling both radical treatment of the tumour and appropriate rehabilitation with hearing aids or cochlear implants [7].

In many cases of VS, hearing preservation surgery remains a challenge, and the mechanisms with which a hearing loss can result are numerous: labyrinthine damage, direct acoustic nerve injury, and cochlear or nerve ischemia [8]. Cochlear ischemia, identified by cochlear response recordings, can occur with different mechanisms such as compression, mechanical damage, and vasospasm. According to some authors, this could be avoided by using vasoactive drugs, even if the benefits are still controversial [8]. Labyrinthine damage can, of course, be prevented by surgical approaches that allow direct visualisation and preservation of the structure, the retrosigmoid approach with retrolabyrinthine meatotomy being the best option for hearing preservation, according to some authors [9,10].

Regarding cochlear nerve integrity, this approach requires identification of the cochlear nerve despite tumour presence, which can be obtained by a good surgical exposure, soft handling of the tumour–nerve interface and, of no less importance, different techniques of cochlear nerve monitoring, which can be used alone or in various combinations [8].

## 2. Materials and Methods

We preliminarily defined our study using the PICO protocol as follows (patient: patients with vestibular schwannoma; intervention: cochlear nerve intraoperative monitoring in hearing-sparing surgery; comparison: absent monitoring, other monitoring; outcome: hearing preservation percentage).

We started by searching the Cochrane library, Scopus, and DynaMed databases without finding any pertinent result.

Then, the PubMed database was systematically screened up to November 2022 using the MESH terms: ((“Hearing”[Mesh]) AND “Monitoring, Intraoperative”[Mesh]) AND “Cochlear Nerve”[Mesh], yielding a total of 20 results. As this method of search appeared too specific, we also used a free term search: intraoperative monitoring AND cochlear nerve, yielding 247 results, in which were included all but 2 pertinent results from the MESH term search. Finally, 15 articles were included in our review.

## 3. Results

**Figure 1:**
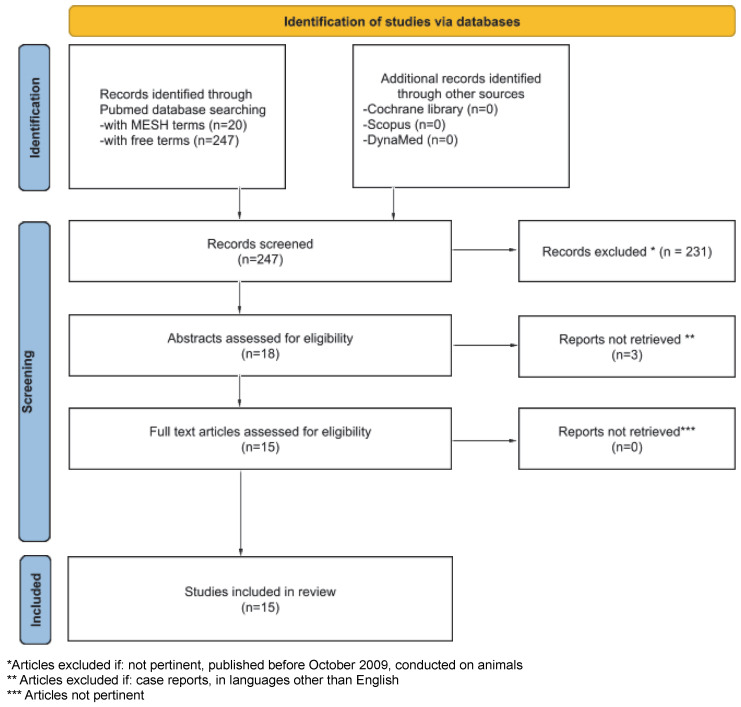
PRISMA flow diagram for literature research (Cochrane library, Scopus and DynaMed) up to November 2022.

In our literature research, among the 245 results we found the systematic review by Youssef et al. [11], dating back to October 2009. We decided to exclude all articles prior to this date and all articles referring to non-hearing-sparing surgical techniques. Starting from this point, a refined search was then conducted on the remaining articles excluding all works prior to 1990, all works conducted on animals, all works in languages other than English, and all works containing less than 1 patient. In the end, 15 articles were included in our review (Figure 1).

Results are summarized in Table 1.

Youssef et al. (2009) conducted a systematic review including a total of four main works, for a total of 423 patients, monitored with either ABRs, CNAPs, or ECochG. Although it emphasized the better functional outcomes of vestibular neuroma surgery, in terms of hearing preservation and facial nerve paresis compared to the past, it did not indicate which technique should be preferred [11].

Simon (2011) considered ABRs, CNAPs, and ECochG in her narrative review. The article concluded that it is crucial to understand the neuro-anatomic and neuro-physiologic principles of all monitoring techniques, in order to choose the better one for each patient [12].

Yamakami et al. (2014), in their retrospective study on 44 patients, concluded that the retrosigmoid approach has excellent functional outcomes when used for small tumor removal if CNAPs, ABRs, and careful surgical technique are used [3].

Ahihara et al. (2014) tested ABRs and CNAPs on their cohort, emphasizing the complementarity of the two techniques [13].

**Table 1:** Characteristics of the included studies in terms of efficacy of the monitoring techniques based on preoperative and postoperative outcomes.

First Author (Year)	Design	Patients (No.)	Monitoring Techniques	VS Size (cm)	Mean Preoperative PTA (dB)	Preoperative SDS	Preoperative WRS	Preoperative ABR	Mean Postoperative PTA (dB)	Postoperative SDS	Postoperative WRS
Yamakami (2009) [5]	?	22	ABRs, CNAPs	≤1.5	32.7	>50%.	?	Yes, preserved	50.8	?	?
Samy Youssef (2009) [11]	SR	423	ABRs, CNAPs, ECochG	Danner et al., 2004 ≤1 to ≥2.5 Piccirillo et al., 2008 ≤1.5 Nedzelski et al., “small” Colletti et al., “small”	?	?	?	?	?	?	?
Simon (2011) [12]	NR	?	ABRs, CNAPs, ECochG	?	?	?	?	?	?	?	?
Yamakami (2014) [3]	RCS	44	ABRs, CNAPs	6 patients “small”: intracanalicular, without APC extension 38 patients “small”: APC extension ≤ 15 mm	*** 19 patients: A 17 patients: B 8 patients: C 0 patients: D	Class A–B PTA ≤ 50	Class A–B WRS ≥ 50	No	*** 5 patients: A 21 patients: B 11 patients: C 7 patients: D	?	?
Aihara (2014) [13]	RCS	121	ABRs, CNAPs	* 35 pz: grade I 38 pz: grade II 40 pz: grade III 8 pz: grade IV	8 pz without preoperative ABR: -Wave I only group: PTA 32 -Flat ABRs group: PTA 37	?	?	Yes, preserved in 113 patients, absent in 8 patients	?	?	?
Zhang (2015) [2]	SR	221	ABRs	>3 (n = 183, 82.8%) <3 (n = 38, 17.2%)	?	?	?	?	?	?	?
Hummel (2016) [14]	PCS	46	ABRs	§ T3A 37%, T3B 35%	61–80	35–10%	?	** 2 patients: class 1 27 patients: class 2 27 patients: class 3 9 patients: class 4 2 patients: class 5	?	?	?
Sun (2018) [15]	RCS	126	ABRs, CNAPs	1.39	36.1	?	83.2%	No	?	?	?
Blandine (2018) [8]	CCS	31 cases, 19 controls	ECochG	* 1 pz: grade I 8 pz: grade II 9 pz: grade III 11 pz: grade IV	*** 15 pz: A 17 pz: B 14 pz: C 4 pz: D	?	?	Yes, absent in 2/3 of patients	*** 8 pz: A 9 pz: B 4 pz: C 29 pz: D	?	?
Mastronardi (2018) [4]	RCS	25	ABRs	2.04 ± 0.9	?	>50%	?	Yes	*** 15 pz: A–B 8 pz: C 2 pz: D	?	?
Matsushima (2019) [16]	?	?	ABRs, CNAPs	?	?	?	?	?	?	?	?
Sass (2019) [17]	RCS	31	CPA master	1.52	36.5	73%	18 pz (58%) >70% 6 pz (19%) 50–70% 7 pz (23%) < 50%	No	59.8	56.4%,	?
Pobożny (2020) [1]	CS	3	CNAPs, ECochG	<2.5	-patient 1: 8 -patient 2: 40 -patient 3: 22	?	-patient 1: 100% 60 dB -patient 2: 80% 100 dB -patient 3: 100% 60 dB	No	-patient 1: 17 -patient 2: 50 -patient 3: anacusia	?	-patient 1: 100% a 65 dB -patient 2: 95% a 80 dB -patient 3 anacusia
Antezana (2021) [18]	CS	2	ABRs, CNAPs	1.8, 2.5	16.6	?	?	No	28.3	?	?
Starnoni (2022) [19]	CS	10	ABRs, PAMR	?	Preop Gardner-Robertson degree 1 = 4 pz 2= 4 pz 3= 2 pz 4= 0 pz	?	?	No	Postop Gardner-Robertson degree 1 = 5 pz 2= 4 pz 3= 1 pz 4= 0 pz	?	?

Question marks (?) indicate unclear or no information provided. Abbreviations: CS case series, CCS case-control study, NR narrative review, SR systematic review, RCS retrospective cohort study, PCS prospective cohort study. * VS grading based on MRI Koos classification; ** ABR classification according to Hannover Classification; *** American Academy of Otolaryngology, Head and Neck Surgery (AAO-HNS) hearing classification system;.§ Hannover classification of tumor extension.

Starnoni et al. (2022) [19] in their cohort of 10 patients, propose PAMRs as a viable cochlear nerve monitoring technique.

Zhang et al. (2015) declared satisfactory results with ABRs cochlear monitoring in their article [2].

Hummel et al. (2016) tested ABRs monitoring, assessing that, especially in the surgical phase, after 60% of tumor removal, their presence has predictive importance on hearing outcome [14].

Sun et al. (2018), in their retrospective review based on 126 patients, stated that dual-modality monitoring using both ABR and CNAP maximized both sensitivity and specificity for the detection of post-surgical hearing decline [15].

Balndine et al. (2018) tested ECochG in a case-control study. This method was found to be effective by these authors, as it effectively detected all surgical events and its intraoperative disappearance warned of poor hearing outcome with no observed false alarm [8].

Mastronardi et al. (2018) tested a new kind of intraoperative ABR by using LS CE-Chirp on 25 patients and, comparing pre- and postoperative ABR results, they stated that it represents a safe and effective method for monitoring the cochlear nerve [4].

Sass et al. (2019), in their retrospective review, tested a new type of monitoring, CPA master, on 31 patients, reporting optimal results [17].

Yamakami et al. (2009) considered ABRs versus CNAPs on a total of 22 patients. They tested a new CNAPs electrode during small tumour removal with a retrosigmoid approach [5]. As CNAPs in their case appear to be unaffected by artefacts during surgical procedures, they are considered more useful than ABRs for cochlear nerve monitoring [5]. Good CNAPs monitoring technique results were also obtained by Antezana at al. (2021), using their new experimental intraoperative electrode, tested on two patients in this article [18].

Much like Ahihara et al. [13], Matsushima et al. (2019) [16] stress the complementarity of ABRs and CNAPs as monitoring techniques.

Pobożny et al. (2020) described their PAMR cochlear nerve monitoring technique, stating pros and cons on a total of 10 patients [1].

We summarized every article setup in Table 1. As shown in this table, we described for each study the design (2 systematic reviews, 1 narrative review, 5 retrospective cohort studies, 1 prospective cohort study, 1 case-control study and 3 case series), the number of patients considered and the different monitoring techniques tested, which, in each study, were either considered alone or in different combinations. The fifth column summarizes the size of VS considered in the different studies and, as can be seen, even if in most cases the radiological KOOS classification was used, not all studies divided the VS according to the same size criteria, arbitrarily setting the cutoff for a small VS. In Table 1 we decided to also include, in order to better underline the efficacy of the different monitoring techniques, whether there were changes from preoperative to postoperative pure tone average (PTA) results, world recognition score (WRS) results, speech discrimination score (SDS) results, and ABR’s pattern.

## 4. Discussion

Table 1 shows the great heterogeneity of data in each study, which was the main limiting factor of this review; this made it challenging to summarize the overall results. Firstly, the studies had different settings, with only 3 being at the top of the evidence pyramid (2 systematic reviews and 1 narrative review) [4,11,12]. The number of patients considered in each study varied greatly, ranging from 423 [11] to 2 [18]; the monitoring techniques also varied greatly. Regarding this point, an important limit was that some studies considered one technique used alone (Zhang et al., Mastronardi et al., Blandine et al., and Hummel et al.) [2,4,8,14], while others, to establish a better monitoring tool, considered different techniques, used in combination or in opposition. Another limit was the heterogeneity of the modality with which each author decided to assess whether a technique tested was to be considered efficient or not. We attempted to standardize the results by extracting reproducible data from all the works, in particular pre- and postoperative PTA, SDS, WRS, and ABR waveforms. The first thing we noticed was that not every study based its results on these objective parameters, and secondly, that even if these outcomes were considered, they were often expressed in different scales, as shown in Table 1. Moreover, even if, in this work, we selected only the studies in which the authors wished for a hearing-sparing surgery, considerations could not be made about the VS size with which every author tested the technique; the scores used were often different, and in some cases absolutely subjective, such as in Youssef et al.’s [11] work, in which the world “small” is simply used to describe the VS size. To sum up, in the 15 studies considered in this review, the monitoring techniques were always different and in different combinations; and the pre- and postoperative parameters considered were always different or expressed in different classification scales. Following is a brief discussion of each test examined.

### 4.1. Auditory Brainstem Responses

Auditory brainstem responses are short-latency, far-field, evoked potentials that reflect the depolarization of several auditory structures of the auditory pathways, from the cochlear nerve to the lower midbrain.

The pattern of this monitoring is composed of seven different waves: [12]

- Wave I, from the proximal part of cochlear nerve VIII;
- Wave II, from cochlear nucleus;
- Wave III, from the lower pons at the level of the superior olivary complex;
- Wave IV, from the mid and upper pons;
- Wave V, from lower midbrain;
- Wave VI, from the medial geniculate body;
- Wave VII, from auditory radiations.

ABRs are generated by sound stimulation of the auditory receptors; the stimulus administered to the patient is a broadband stimulus called click, which is mainly determined by the ear’s sensitivity to frequencies above 2000 Hz [13]. Recording is done at distance from their actual generators, through electrodes placed on the scalp, making these potentials so called “far-field” [12].

The most useful information in cochlear nerve neuromonitoring with ABRs is delivered by waves I and V, which are the most stable; cochlear nerve damage results in a delay in latency and a reduction in the amplitude of wave V [13].

To a lesser extent, waves II and III could be considered, noting that they could vary somewhat during the monitoring and do not have direct clinical significance. Waves IV, VI, and VII are highly variable and thus have no role in intraoperative neurophysiology. When approaching this technique, it is crucial to obtain a patient’s waveform before surgery, as in many individuals, waves III and V could be absent [8].

Regarding its major limitations, compared to other techniques, such as cochlear monitoring, a large number of samples (up to several hundred repetitions) are needed to achieve optimal nerve monitoring with a clear identification of wave alterations at a given stimulus level [1,8]. Moreover, the poor signal-to-noise ratio of ABR necessitates a long data acquisition time to achieve an adequate signal-to-noise ratio, and it cannot provide real time results [1,5]. Being a far-field recorded potential, it is susceptible to various artefacts, including those from the electrical network, general anesthesia, body temperature, dural opening, saline irrigation of surgical field, surgical microscope, high-speed drill, and ultrasonic aspirator [1,5,19].

Critical ABR pattern alteration usually occurs during the most delicate parts of surgery, namely during dissection in the internal auditory canal, or during direct manipulation of the cochlear nerve. This deterioration must be identified and immediately avoided by changing surgical strategy or by performing sufficient breaks to enable ABR recovery [14].

Regarding the monitoring of the first part of the auditory pathway, it has been pointed out by some authors that other techniques should be preferred, such as cochlear monitoring. In fact, ABR wave I is smaller than its cochlear action potential counterpart; if not correctly identified, it could fail to signal problems in the distal auditory pathway, such as cochlear ischemia and cochlear nerve damage [8]. In fact, if compared to CNAPs, this has the advantage of being a near-field technique, with a higher chance of estimating cochlear nerve damage during surgery, and it could even happen that click-evoked CNAPs are detected during surgery in patients without apparent waves in ABR [13].

Some authors tried to overcome the limits of ABRs by using different kinds of stimulation. Mastronardi et al., instead of using clicks, tried using LS-CE-Chirp^®^; unlike click stimulus, using LS-CEChirp^®^ allows faster surgeon alerting, around 10–15 s from when a variation of conduction parameters of acoustic pathway is detected, and the morphology of wave V appears to be more stable [4].

ABR wave V and CNAP N1 amplitude changes are associated with distinct changes in postoperative pure tone average (PTA) and world recognition score (WRS) in most cases [15]. Nonetheless, in some cases, ABR preservation at the end of the monitoring session may not mean hearing preservation, while its loss, which only means loss of synchronous responses of brainstem neurons to short sound stimuli, may happen despite serviceable postoperative hearing [5,8]. Some authors claim that one should confirm the state of wave V using alternative sound-evoked ABR before surgery, as the normal latency and amplitude may be different from those on click-evoked ABR [13]. Those same authors recommend that ABR should be recorded by stimulation of both sides before surgery, and the latency of wave V or I–V interpeak latency on both sides should be noted [13].

Even though we previously described ABR limits, one should not forget that its low invasiveness and well-known underlying neural mechanisms often make this technique the most appreciated one in operating theatres around the world.

### 4.2. Direct Eighth Cranial Nerve Monitoring

Direct eighth cranial nerve monitoring (DENM) is a new system for intraoperative, continuous, near-real-time monitoring of cochlear nerve function, described by Sass et al. [17]. It uses continuous acoustic stimulation in the ear canal, recording the evoked potentials from the dorsal cochlear nucleus using a specially designed electrode placed directly upon the brainstem, in Luschka’s foramen. This electrode rests upon the dorsal cochlear nucleus of the brainstem, with practically real-time monitoring of cochlear nerve function. It provides the option to immediately change the tumor dissection technique when the nerve suffers, as opposed to other techniques, such as ABR-based systems, in which there is a functional delay of up to 30 to 60 s. Moreover, direct eighth cranial nerve monitoring, allows intraoperative mapping and thus the precise localization of the cochlear nerve in its entire trajectory, from the brainstem to the fundus of the internal auditory canal [17].

Sass et al. demonstrated that with this new near real-time cochlear nerve neuromonitoring system any hearing can be preserved in 83% of cases, and serviceable hearing can be preserved in 77% of patients with a growing vestibular schwannoma who underwent surgical removal through a modified, extended retrolabyrinthine approach [17].

The limits of this technique include the need to place the probe before tumor dissection, as well as probe displacement during surgery. Furthermore, securing the probe on the nerve can cause iatrogenic damage, and accumulating cerebrospinal fluid can alter responses [17].

DENM is a new monitoring system with promising results, although the literature regarding its actual pros and cons still seems to be insufficient.

### 4.3. Cochlear Compound Nerve Action Potentials

Cochlear compound nerve action potential (CNAP) is a monitoring technique that uses an electrode positioned by the surgeon proximal to the site of surgery and directly on the nerve. It allows continuous intraoperative live monitoring of the function of the acoustic nerve by summing all the nerve action potentials arising in its the nerve fibers [12]. The electrode being so close to the mass, tumor size and the size of the access via the middle cranial fossa may influence the possible location of the measurement electrode [1]. Its benefits are most significant in cases of small- or medium-sized tumors; in fact, because of the particular position in which the electrode must be place, this technique is only applicable in cases in which at least some portion of the nerve is not covered by the mass, so it is not feasible in cases of large vestibular schwannomas [19].

Compared to other techniques, such as ABRs, these evoked responses have higher amplitudes, being near-field recording techniques, and are more advantageous to use in noisy environments or in those patients in which preoperative ABR waves are not present. Moreover, they are deemed more stable in some situations, such as drilling and manipulation of the surgical site [12].

When a lack of response is observed, it reveals the lack of potential generation in the spiral ganglion following acoustic stimulation, usually caused by prolonged disruption of inner ear perfusion, which may result from damage to the labyrinthine artery or, less frequently, the anterior inferior cerebellar artery [12].

On the contrary, lowered amplitudes and extended latencies of potentials usually indicate a reduction in the number of neurons and nerve fibers as well as the disruption of stimulation and response obtained from the neurons of the spiral ganglion [1].

The main disadvantage of this technique derives from the need to place the electrode directly on the acoustic nerve; it requires careful placement from the surgeon and steady maintenance during surgery, at the same time avoiding iatrogenic damage of the nerve fibers, balancing the need to monitor and the need to have a clear view of the surgical field. It is not uncommon for this electrode to shift position during surgical manipulation, resulting in false-positive changes of the recorded potentials [12].

To overcome this problem, promising technical advancements have been made by Yamakami et al., by designing a new intracranial electrode for CNAP recording in which a small tuft of cotton is secured on the tip of a fine, malleable urethane-coated wire, making the electrode itself more stable and less traumatic on the acoustic nerve [5].

Moreover, this technique is limited by the fact that the surgeon needs early access to the brainstem and eighth cranial nerve entry zone to position the electrode, and this generally limits its application to smaller tumors [18].

Used alone, this technique is not deemed optimal for intraoperative nerve monitoring. On the contrary, if associated with other near-field, short-latency techniques such as ECochG, CNAPs could provide information from various sites of the auditory pathway both distally to the tumor (the distal segment of the vestibulocochlear nerve—TT-ECochG) and proximally to the tumor (the proximal part of the cochlear nerve—direct CNAP) [1].

If used in combination with ABR instead, it provides a continuous and stable assessment of hearing function [16] with, in some studies, up to 83% sensitivity and 100% specificity in predicting postoperative hearing decline [15]. In fact, CNAPs are useful for guiding real-time tumor dissection, while an intact postoperative ABR waveform provides a high degree of prognostic confidence that at least some hearing has been preserved [15].

### 4.4. Transtympanic Electrocochleography

Transtympanic electrocochleography (ECochG) is a monitoring technique that, using tone bursts condensation and rarefaction, provides an almost real-time near-field response from the peripheral segment of the vestibulocochlear nerve and the cochlea, corresponding to wave I of the ABRs.

It requires insertion of an electrode through the tympanic membrane on the promontory bone, even if extratympanic ECochG has also been described. The latter, in fact, does not require a myringotomy but only surface electrodes and, according to some authors, it may provide similar results [12].

Regarding its efficacy in cochlear nerve monitoring during surgery, it must be kept in mind that, although the response from the nerve is practically real-time, it only involves the first segment, resulting in a far from optimal hearing-monitoring technique, if used alone [1]. Moreover, its signal, during surgery, is not always reliable in terms of wave morphology, amplitude, and/or latency. In fact, it has been observed that surgical manipulation of the region disturbs the signal; in particular it has not been deemed reliable in the case of drilling or bipolar coagulation in the proximity of the surgical site, or in the case of nerve stretching, these being, in fact, the conditions in which accurate monitoring of the nerve fibers are essential [1], even if not all authors agree on this point [12].

According to other authors, there are indeed some advantages to this technique. In fact, compared to far-field recorded ABRs, it offers higher amplitude potentials, monitoring the function of the cochlea and the very origin of the acoustic nerve segment, potentials of which are difficult to record with distant electrodes. As a consequence, for certain types of procedures involving possible damage only to the inner ear and/or cochlea, such as middle ear reconstructive surgeries, ECochG monitoring could be the monitoring of choice, keeping in mind that if a clean dissection of the cochlea from the brain stem occurs, ECochG will not be able to detect the damage, since their recordings are still in place, although an ABR waveform will evidence lack of the last waves [12].

All things considered, almost every author agrees that although ECochG could be a valid help during inner ear surgery, it is not safe to use it alone, but it is always best to integrate the information it can provide with that acquired by other complementary techniques, such as ABRs or cochlear compound nerve action potentials.

### 4.5. Distortion Product Otoacoustic Emissions

Distortion product otoacoustic emissions (DPOAEs) are faint, low-level sounds resulting from the movement of cochlear hair cells in response to a pair of pure tones at neighboring frequencies. DPOAE level depends on outer hair cell integrity and on its ability to amplify sound [8]. It is used for intraoperative cochlear nerve monitoring only anecdotally, although many animal studies have been performed [12]. DPOAEs appear to be very sensitive to cochlear ischemia, having the potential to become a sensitive tool for early identification (earlier than with ECochG) of a decrease in cochlear blood flow [12].

### 4.6. Postauricular Muscle Responses

Postauricular muscle responses (PAMRs) are large sound-evoked muscle action potentials elicited by monaural or binaural stimulation and are extremely variable across the population and within individuals. They are categorized as auditory evoked vestigial muscular responses and act by pulling the ear upward and backward to help localize the source of the sound. The receptor organ driving PAMRs is the cochlea.

They have been recently considered in a work by Starnoni et al. (2022) [19] as a possible technique for cochlear nerve monitoring in hearing-sparing surgery.

PAMRs are measured with surface electrodes placed on the skin at the midpoint of the postauricular muscle and on the back of the pinna, even if the “classical” electrode setting used for BAEPs could also be used. The auditory stimuli must be loud (at least 80 dB) and the waveform obtained consists of two peaks, a negative peak between 10 and 15 msec and a positive peak between 15 and 18 msec, with amplitudes between 10 and 100 µV.

The muscular responses obtained after sound stimulation are often much larger than the BAEPs; in addition, compared to the latter, the waveform obtainable is more stable and has a shorter latency due to their higher signal-to-noise ratio.

Starnoni et al. [19] proposed this technique in a cohort of 10 patients with large vestibular schwannomas and preoperative serviceable hearing, the limited number of their cohort being the major limit of this study.

The correct setting of stimulation intensity is essential to obtain a good cochlear response without adjacent nerve stimulation, with the most reliable stimulation intensity being 1 mA at 1 Hz.

In addition, the waveform obtained must be cleaned of disturbing electrical signals, such as those belonging to the startle reflexes group (including the auditory blink reflex, which is the bilateral contraction of the orbicularis oculi muscle after an audio stimulation of over 80 dB), which share some common pathways with PAMR but which can be ruled out based on its longer latency and by monitoring the absence of muscular contraction in muscles responding to startle reflexes (orbicularis oculi, masseter, trapezius…).

The major limit of this technique concerns the fact that it provides an intermittent mapping of the position and trajectory of the cochlear nerve. Thus, compared to continuous monitoring techniques, it has to be performed several times during surgery in order to successfully preserve cochlear nerve function. Moreover, PAMRs are strongly influenced by muscle-blocking agents used in general anesthesia.

On the contrary, PAMR has the advantage of being able to map the entire trajectory of the cochlear nerve on the tumor capsule without dissecting between the capsule and the nerve. If elicited during surgery, PAMRs allows the identification of the entire cisternal course of the cochlear nerve, enabling preservation of neural function.

PAMR is a controversial new cochlear nerve monitoring technique. It has advantages and disadvantages, but it is always recommended that it be used in association with other well-studied methods, such as ABRs, that can continuously monitor the integrity of the entire hearing pathway [19].

## 5. Conclusions

This review attempted to sum up the existing literature regarding cochlear nerve monitoring in hearing preservation surgery. Although other types of nerve monitoring, such as facial nerve monitoring, have been proven to be needed and effective, there is still a lot of confusion regarding cochlear nerve intraoperative monitoring and its clinical repercussions. Different works have been published, but they are all heterogeneous, with different pre- and postoperative outcomes considered, and with different techniques included, either alone, or in comparison to each other. Our impression is that the perfect monitoring technique does not exist. Each has many pros and cons, so the clinician, based on surgical parameters such as vestibular schwannoma dimension and location, the status of preoperative hearing, and the most adequate surgical technique, should choose different kinds of monitoring that are complementary to each other. CNAPs associated with ABRs should provide real-time intraoperative monitoring with stable and very well-known wave patterns comprising the whole auditory pathway, even if some authors praise a single technique used alone, such as in the case of Yamakami et al., with the near-field monitoring technique using their new electrode [5].

Further, more standardized studies are needed to assess the real efficacy of the different techniques and to determine which one is the best when it comes to the goal of complete removal of the tumor along with serviceable postoperative hearing.

## Data Availability

Not applicable.
